# Supplementary material for: Effect of sea surface temperature in El Niño regions on dengue dynamics in Colombia: Evidence from causal machine learning
Source: PLOS Glob Public Health. 2026 Jan 23;6(1):e0005796. doi: 10.1371/journal.pgph.0005796 (PMC12829929; doi:10.1371/journal.pgph.0005796)
Supplement: Attachment — Submitted filename: Response to reviewers.docx [file pgph.0005796.s003.docx]

Journal Requirements:

1. Please update your online Competing Interests statement. If you have no competing interests to declare, please state: “The authors have declared that no competing interests exist.”

R: We accepted the comment, and we have updated the online Competing Interests statement

2. Please provide separate main figure files in .tif or .eps format only and remove any figures embedded in your manuscript file. Please also ensure that all files are under our size limit of 10MB. Please leave the figure captions in the manuscript.

For more information about how to convert your figure files please see our guidelines: https://journals.plos.org/globalpublichealth/s/figures

R: We accepted the comment, and we have provided separate figure files in .tif format and removed figures embedded in the manuscript file.

3. Please ensure that you refer to Figure 5 in your text as, if accepted, production will need this reference to link the reader to the figure.

R: We accepted the comment, and we have referred to Figure 5 in the text (lines 389-395).

4. Some material included in your submission may be copyrighted. According to PLOS’s copyright policy, authors who use figures or other material (e.g., graphics, clipart, maps) from another author or copyright holder must demonstrate or obtain permission to publish this material under the Creative Commons Attribution 4.0 International (CC BY 4.0) License used by PLOS journals. Please closely review the details of PLOS’s copyright requirements here: PLOS Licenses and Copyright. If you need to request permissions from a copyright holder, you may use PLOS's Copyright Content Permission form.

Please respond directly to this email or email the journal office and provide any known details concerning your material's license terms and permissions required for reuse, even if you have not yet obtained copyright permissions or are unsure of your material's copyright compatibility.

Potential Copyright Issues:

Figure 1: Please confirm whether you drew the images / clip-art within the figure panels by hand. If you did not draw the images, please provide (a) a link to the source of the images or icons and their license / terms of use; or (b) written permission from the copyright holder to publish the images or icons under our CC-BY 4.0 license. Alternatively, you may replace the images with open source alternatives. See these open source resources you may use to replace images / clip-art:

- https://commons.wikimedia.org

- https://openclipart.org/

R: We accepted the comment, and we have changed the icons of Figure 1 to use icons from openclipart.org, and provided the link to the source of the icons and their terms of use.

5. If the reviewer comments include a recommendation to cite specific previously published works, please review and evaluate these publications to determine whether they are relevant and should be cited. There is no requirement to cite these works unless the editor has indicated otherwise.

6. Please review your reference list to ensure that it is complete and correct. If you have cited papers that have been retracted, please include the rationale for doing so in the manuscript text, or remove these references and replace them with relevant current references. Any changes to the reference list should be mentioned in the rebuttal letter that accompanies your revised manuscript. If you need to cite a retracted article, indicate the article’s retracted status in the References list and also include a citation and full reference for the retraction notice.

R: We accepted the comment, and we have reviewed the reference list to ensure that it is complete and correct.

**Comments to the Author**

1. Does this manuscript meet PLOS Global Public Health’s [publication criteria](https://track.editorialmanager.com/CL0/https:%2F%2Fjournals.plos.org%2Fglobalpublichealth%2Fs%2Fcriteria-for-publication/1/010f019b2a48cf4f-850e8a47-5e43-45c7-acca-5402479c48aa-000000/6_Jmss5rlvc-wwaBwaZ3hfNC3VEyMOByJEDD0m8_Lx4=242)? Is the manuscript technically sound, and do the data support the conclusions? The manuscript must describe methodologically and ethically rigorous research with conclusions that are appropriately drawn based on the data presented.

Reviewer #1: Yes

2. Has the statistical analysis been performed appropriately and rigorously?

Reviewer #1: Yes

3. Have the authors made all data underlying the findings in their manuscript fully available (please refer to the Data Availability Statement at the start of the manuscript PDF file)?

The [PLOS Data policy](https://track.editorialmanager.com/CL0/https:%2F%2Fjournals.plos.org%2Fglobalpublichealth%2Fs%2Fdata-availability/1/010f019b2a48cf4f-850e8a47-5e43-45c7-acca-5402479c48aa-000000/0cecWBS82yoCu2ganrFGYtSgrUafVJSLbWYJWFFktwo=242) requires authors to make all data underlying the findings described in their manuscript fully available without restriction, with rare exception. The data should be provided as part of the manuscript or its supporting information, or deposited to a public repository. For example, in addition to summary statistics, the data points behind means, medians and variance measures should be available. If there are restrictions on publicly sharing data—e.g. participant privacy or use of data from a third party—those must be specified.

Reviewer #1: Yes

4. Is the manuscript presented in an intelligible fashion and written in standard English?

PLOS Global Public Health does not copyedit accepted manuscripts, so the language in submitted articles must be clear, correct, and unambiguous. Any typographical or grammatical errors should be corrected at revision, so please note any specific errors here.

Reviewer #1: Yes

5. Review Comments to the Author

Please use the space provided to explain your answers to the questions above. You may also include additional comments for the author, including concerns about dual publication, research ethics, or publication ethics. (Please upload your review as an attachment if it exceeds 20,000 characters)

Reviewer #1: Dear Authors,

Thank you for submitting your manuscript “Effect of sea surface temperature in El Niño regions on dengue dynamics in Colombia: Evidence from causal machine learning” to PLOS Global Public Health.

Overall, this is a well-structured and rigorous study addressing an important topic at the intersection of climate science, epidemiology, and causal machine learning. The manuscript is clearly written, the methodological framework is strong, and the results are well presented. Your use of DML, DAG-based adjustment, and robustness tests demonstrates methodological sophistication.

My final recommendation is Accept after Minor Revision. Below, I detail both your already-addressed comments and several additional minor improvements that would strengthen clarity, transparency, and reproducibility.

General Assessment

Strengths

The study uses a large temporal and spatial dataset (1,044 municipalities, 2013–2023).

The causal machine learning approach is innovative relative to most ENSO–dengue literature.

The robustness tests offer valuable insights about remaining bias.

The manuscript is detailed and transparent, especially in the Methods section.

Figures are clear and generally well designed.

Areas for Minor Improvement

While the paper is strong, a few minor issues require clarification or polishing to increase readability and rigor. These are not major scientific concerns, but they will improve the manuscript's overall quality.

Specific Minor Revisions

1. Improve the flow and clarity of the Introduction

The Introduction is long and very method-heavy. Consider reorganizing:

Start with the dengue–climate relationship.

Then discuss SST and ENSO relevance.

Then introduce the novelty of causal ML approaches.

Some references appear duplicated (e.g., ENSO–dengue relationships). Confirm consistency.

R: We accepted the comment, and we have improved the Introduction and confirmed the consistency of references (lines 52-91).

2. Provide clearer justification for excluding altitude > 2,200 m

You mention the threshold, but the Methods would benefit from a short sentence referencing biological limits of *Aedes aegypti*, or citing a specific study validating this elevation threshold.

R: We accepted the comment, and we have included a justification sentence for the altitudinal threshold of 2,200 m, and included a reference (lines 109-114).

3. Improve Table 1 description

Add a sentence explaining the role of each index more clearly (e.g., why North Atlantic SST is relevant as a confounder). This helps non-climate-experts.

R: We accepted the comment, and we have included a new paragraph explaining the role of the atmospheric and oceanic indices of Table 1 (lines 130-137).

4. Clarify the collider reasoning

You identified MPI, rainfall, temperature, etc. as colliders and excluded them to avoid collider bias.

This is excellent but unusual for most readers; please: briefly explain the intuition (e.g., “conditioning on variables influenced jointly by exposure and confounders induces bias”). cite a standard causal inference reference.

R: We accepted the comment, and we have included new sentences to clarify the collider reasoning and include two standard causal inference references (lines 358-366).

5. Expand on the potential mechanism behind altitude-related CATE

You correctly state that the pattern is “not clear”. I recommend adding: either possible hypothesis (vector biology, indoor–outdoor behavior, socio-economic gradients), or stating more explicitly that this should be part of future work.

R: We accepted the comment, and we have included a new sentence describing our hypothesis about the mechanism behind the altitude-related CATE (lines 429-436).

6. Improve the explanation of the negative control exposure. The description is technically correct, but consider adding: one sentence describing why “future SST cannot affect past dengue cases.”

R: We accepted the comment, and we have modified the paragraph to improve the explanation of the negative control exposure (lines 277-281).

7. Figures – enhance labeling

Figure 4: Add exact SD values in the caption for each El Niño region.

R: We accepted the comment, and we have modified Figure 4 to add the SD values for each El Niño region.

Figure 5: Indicate the altitude range in the figure (e.g., min–max).

R: Please note that the X-axis of Figure 5 indicates the altitude in meters of the Colombian municipalities included in the study (i.e., min approximately 0 m and max 2,200 m).

8. Clarify limitations related to passive surveillance

The limitations section mentions SIVIGILA constraints, but it would help to emphasize variability in laboratory confirmation rates between years. Add one sentence about underreporting during healthcare disruptions.

R: We accepted the comment, and we have modified the paragraph to clarify limitations related to passive surveillance (lines 453-459).

9. Discussion – reduce repetition

The discussion repeats the ENSO–dengue relationship several times. Consider merging overlapping paragraphs for conciseness.

R: We accepted the comment, and we have modified the Discussion section to reduce repetition (lines 423-512).

10. Typographical and minor language edits

Examples:

Line 169: “avoiding the induction of other forms of bias” → “to avoid inducing other forms of bias.”

R: We accepted the comment, and we have modified the sentence (lines 169-170)

Line 422: “not clear what the mechanism…” → “the underlying mechanism remains unclear.”

R: We accepted the comment, and we have modified the sentence (lines 429)

Check spacing inconsistencies around parentheses and units.

R: We accepted the comment, and we have checked spacing inconsistencies.

Optional but Helpful Additions

These are optional, but could improve the manuscript:

Add a short data availability note within the Results section referencing the GitHub repository.

R: We accepted the comment, and we have added a short note referencing the GitHub repository (lines 420-421)

Include a map of Colombia showing municipalities included/excluded after altitude filtering.

R: We accepted the comment, and we have modified Figure 2 to include a map of Colombia municipalities included/excluded after altitude filtering.

Add citations related to SST impacts on ocean–climate teleconnections in Latin America.

R: We accepted the comment, and we have modified the paragraph to add citations related to SST impacts on ocean–climate teleconnections in Latin America (lines 63-64).

Add this recent study to your references: Shabani, F., Raie, M., & Kabiri, K. (2025). Studying the Impact of Sea Surface Temperature (SST) Changes and El Niño Phenomenon on Coral Reefs Bleaching around Kish Island in the Northern Persian Gulf: A Remote Sensing Approach. Deep Sea Research Part II: Topical Studies in Oceanography, 105550.

Overall

Your paper is scientifically valuable and nearly ready for publication. I am confident that addressing these minor points will strengthen the manuscript further.

Recommendation: Accept after Minor Revision.
